# Why and When Team Reflexivity Contributes to Team Performance: A Moderated Mediation Model

**DOI:** 10.3389/fpsyg.2019.03044

**Published:** 2020-01-21

**Authors:** Mengxi Yang, Hilary Schloemer, Zheng Zhu, Yuying Lin, Wansi Chen, Niannian Dong

**Affiliations:** ^1^School of Economics and Management, Tsinghua University, Beijing, China; ^2^Neil Griffin College of Business, Arkansas State University, Arkansas, AR, United States; ^3^Business School, Renmin University of China, Beijing, China; ^4^Department of Business Administration, School of Business, East China University of Science and Technology, Shanghai, China; ^5^Donlinks School of Economics and Management, University of Science and Technology Beijing, Beijing, China

**Keywords:** team reflexivity, team diversity, team decision quality, team performance, information/decision perspective

## Abstract

Team reflexivity has gained popularity as a phenomenon of interest in team research, but mixed theorizing around the relationship between team reflexivity and team performance indicates that the relationship is not fully understood. In an effort to improve our understanding and explain why and when team reflexivity will be conducive to team performance, we examine the role of team diversity as a possible boundary condition and of team decision quality as an explanatory mechanism. Using survey data from 82 teams with 82 leaders and 194 team members, we find that team decision quality is a partial mediator of the relationship between team reflexivity and team performance and that team diversity strengthens this mediating relationship. We also find that team diversity moderates the relationship between team reflexivity and decision quality. Taken together, these findings suggest that reflexivity is most effective in conditions of informational richness, such as when teams have high diversity, as the reflective process allows team members to capitalize on their varied perspectives to improve the quality of their decisions and, thus, their performance.

## Introduction

As teamwork has become an indelible part of the modern workplace, much effort has been made to examine the processes and conditions that support team performance. Teams can be conceptualized as information-processing systems, drawing information from members and the environment to analyze and develop solutions for complex problems, and, as such, processing information is an essential part of most teamwork (Schippers et al., 2014). Building on this conceptualization, one stream of research has focused on team reflexivity, the extent to which team members collectively reflect upon the team’s objectives, strategies, and processes, and adapt them to complex and unpredictable circumstances as needed (West, 1996), as a contributor to team performance. This conscious and critical reflection has been generally regarded as a positive contributor to team performance, as it allows for learning, informational exchange, and intentional, incremental improvement; however, there is an inherent cost in time and energy for this reflective process, as well as the potential for conflict resulting from the critique (Moreland and McMinn, 2010). This cost may, at times, mitigate the performance benefits of team reflexivity (Schippers et al., 2013).

Recent work has attempted to unearth the mechanisms and boundary conditions of the positive effects of team reflexivity on team performance in order to better understand the phenomenon and offer better recommendations to those managing teams. This work has focused on exploring conditions in and mechanisms through which it makes theoretical sense for an intentionally reflective process like team reflexivity to be beneficial for team performance. For example, by drawing on a learning perspective, Schippers et al. (2013) found that teams only benefited from reflective processes when they had lower levels of initial performance and that the benefit was derived from increases in team learning. Others have taken an information-processing perspective, focusing on the role of team reflexivity in creating shared task representations that allow team members to work together more effectively (van Ginkel and van Knippenberg, 2009; van Ginkel et al., 2009).

We expand on these efforts to develop a better understanding of the team reflexivity-performance relationship by examining the role of team diversity and team decision quality. Drawing on the conceptualization of teams as information-processing systems (Schippers et al., 2014), we argue that the effect of team reflexivity on team performance is partially explained by its positive effect on team decision quality. Because team reflexivity requires systematic information processing, focusing team members’ attention on reflecting on past task performance and adjusting future task goals and plans to account for inter- and intra-team conditions (West, 1996), we expect team reflexivity to promote high quality information-processing and to reduce information processing failures. Thus, we examine team decision quality as a proximal outcome of team reflexivity that, in turn, contributes to team performance.

**FIGURE 1:**
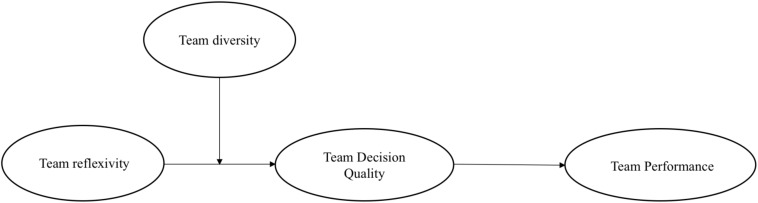
Conceptual model.

Team decision quality is, in part, a function of the quality of information used to make team decisions (Carmeli and Schaubroeck, 2006), underlining the importance of information itself as well as information sharing and processing within teams. Building on this idea, we employ the information/decision-making perspective of diversity (Williams and O’Reilly, 1998; van Knippenberg et al., 2004) to examine whether the heterogeneous information present in diverse teams influences the strength of the team reflexivity-performance relationship. This perspective suggests that higher levels of diversity create the need for groups to “reconcile conflicting viewpoints… [and] more thoroughly process task-relevant information,” increasing their performance (van Knippenberg et al., 2004, p. 1009). We argue that, because of this need, teams with higher diversity will benefit more from intentional, collective reflection on their performance and processes, allowing diversity to strengthen the indirect effect of team reflexivity on team performance through team decision quality (see Figure 1 for conceptual model).

Taken together, we seek to contribute to the reflexivity literature by integrating the information-processing and team diversity literatures to explore why and when team reflexivity contributes to team performance. We build on recent work examining the effect of cognitive processes in the reflexivity-performance processes (e.g., van Ginkel and van Knippenberg, 2009; Schippers et al., 2013) and recent efforts to establish the boundary conditions within which reflexivity has a positive effect on team performance (e.g., Schippers et al., 2013, 2015). To do this, we first examine a new mechanism, team decision quality, to examine why team reflexivity is related to team performance. Then we test an important boundary condition of team reflexivity, team diversity. Specifically, we examine how team reflexivity interacts with team diversity to influence team performance and find that reflexivity within more diverse teams can engender better performance through the effect on team decision quality. By examining a mediator and moderator, we aim offer a clearer understanding of the nature of the team reflexivity-performance relationship and the circumstances under which managers can expect reflective processes to bolster team performance. We further contribute to the reflexivity literature by answering the call for more evidence to explain the conflicting perspectives on the team reflexivity-performance relationship (e.g., Moreland and McMinn, 2010).

## Theoretical Foundations and Hypothesis Development

### Team Reflexivity and Team Performance: The Mediating Role of Decision Quality

Team reflexivity refers to the extent to which group members reflect on and communicate about the group’s objectives, strategies, and processes, allowing them to interpret their accomplishments and prepare for future action (West, 1996). Given that team reflexivity involves the review of prior experience and consideration of future development, team reflexivity has the potential to help teams engage in more beneficial interactions with team members (e.g., information sharing), identify inter/intra-team conditions that affect their performance (e.g., problems, opportunities, challenges), and enhance understanding of team goals (West, 1996). This collective adjustment of thoughts and actions occurs through four main processes: feedback seeking from peers, reflection and self-explanation, data verification, and planning (Chen et al., 2018). These processes can allow team members to collect and process the feedback from their peers’ experience and their own experience. By seeking feedback from coworkers and collecting cross-validated information, teams can develop a more comprehensive understanding of their prior successes and failures and then carefully consider the appropriate approach to future tasks (West, 1996).

To be specific, the team reflexivity process begins with team members seeking feedback from peers (Ellis and Davidi, 2005). Then, at the core phase of team reflexivity, reflection and self-explanation, team members are encouraged to draw on individual and collective experiences from prior successes and failures to explain their previous performance outcomes, laying the foundation for identifying inter/intra-team conditions, understanding team goals, and analyzing opportunities and challenges (Ellis and Davidi, 2005; Tannenbaum and Cerasoli, 2013). By sharing and reviewing past experiences, team members can verify information and perceptions (i.e., by cross-validating information across team members; Ellis et al., 2010) and finish the reflexivity process by discussing action plans for future tasks that include a shared understanding of the tasks and appropriate ways to accomplish them (van Ginkel et al., 2009). Generally speaking, team reflexivity creates conditions under which team members can apply summarized knowledge and skills from previous experiences to cope with their future tasks and challenges (Schippers et al., 2015).

This process has the potential to contribute positively to team effectiveness as it allows them to consciously and iteratively improve their performance. Significant empirical work suggests that team reflexivity can contribute positively to team performance generally (e.g., Schippers et al., 2007, 2018a,b; van Ginkel and van Knippenberg, 2009; Otte et al., 2018) as well as employee satisfaction, commitment (Schippers et al., 2003), and innovation (Schippers et al., 2015). Similarly, a meta-analysis demonstrated that team debriefings, a more general reflective process, can enhance team performance (Tannenbaum and Cerasoli, 2013). However, some have suggested that the relationship between team reflexivity and performance is not so straightforward, as this process has the potential to tie up team members’ time and cognitive resources in a way that is detrimental to performance (Moreland and McMinn, 2010; Schippers et al., 2013). Additionally, some limited empirical work has found support for a negative or null effect of team reflexivity on performance under some conditions (e.g., Brav et al., 2009; Schippers et al., 2013).

In an effort to examine the complexities of this relationship, we first examine the mediating mechanism of team decision quality. Team decision quality refers to the extent to which decisions made by the team meet the team’s goals, are based on the best available information and valid argumentation, and can contribute to overall team effectiveness (Dooley and Fryxell, 1999; Carmeli and Schaubroeck, 2006). Because reflexivity is a process of collecting, exchanging, and integrating information, it can improve the information available for decision-making and improve team decision quality. Decision quality is one element that can influence performance (e.g., Carmeli and Schaubroeck, 2006), but performance is a complex phenomenon that is also influenced by other variables, potentially including team reflexivity. Thus, decision quality can function as a more direct and proximate outcome of team reflexivity than team performance and as an explanatory mechanism for the team reflexivity-team performance relationship. Better understanding of the mechanism of effect for team reflexivity allows for more precise theorizing and testing around the complexities of the reflexivity-performance relationship. Specifically, identifying this more proximate outcome of team reflexivity allows us to begin to determine whether the previous mixed results can be explained by exogenous effects on team performance or on the team reflexivity process itself.

The four phases of team reflexivity (i.e., feedback-seeking from peers, reflection and self-explanation, data verification, and planning; Chen et al., 2018) center on information. Based on information from their peers’ feedback and their own experience, team members engage in reflection and self-explanation, and efforts to cross-validate information can improve information sharing and accuracy (DeChurch and Mesmer-Magnus, 2010). These processes can contribute to the formulation of a better plan, which in turn, promotes team performance (DeShon et al., 2004). Thus, we argue that the process of team reflexivity centers on exchanging, elaborating, and integrating information to make high-quality decisions. In support of this contention, work has shown that integrating distributed information within teams allows teams to make higher quality decisions (De Dreu et al., 2008).

To be specific, team reflexivity encourages team members to engage in systematic information processing (De Dreu, 2007), in which individuals evaluate information deeply and elaborately (Chaiken and Trope, 1999), and reduces information processing failures (Schippers et al., 2014). Studies have suggested that systematic information processing can help teams overcome information sampling bias and, therefore, make better decisions (De Dreu, 2007; Schippers et al., 2014). For instance, consideration and discussion of distributed information, that which is not possessed by all team members, has been found to contribute to high-quality decisions (Postmes et al., 2001; Scholten et al., 2007). In contrast, individuals might engage in shallow information processing and jump to conclusions when sufficient evidence is absent (Lerner and Tetlock, 1999). Thus, we argue that team reflexivity contributes to team decision quality.

Hypothesis 1: Team reflexivity is positively related to team decision quality.

A high quality of decision-making can prepare teams for future success. High decision quality originates from members’ concentration on the content and cognitive meaning of a message, open communication of heterogeneously distributed information, and full exploration of ideas (Dooley and Fryxell, 1999). Thus, high quality decision-making means that team members can leverage valid and comprehensive information to develop future plans and strategies to optimally align team actions to environmental demands (Dean and Sharfman, 1996). Under such conditions, team members establish and understand and optimal course of action, and they feel confident and may be motivated to implement team decisions and. in turn, achieve better team performance. Following this logic, we argue:

Hypothesis 2: Team decision quality mediates the relationship between team reflexivity and team performance.

### The Moderating Effect of Team Diversity

As discussed previously, team reflexivity can be viewed as systematic information processing that sets the stage for better team performance. However, information quality is a necessary, though not sufficient, condition for systematic information processing to contribute to performance and decision quality – if the informational inputs into the process are of poor quality, the outputs will not realize many of the benefits of the systematic process. As we argue below, when decision-making teams have low diversity, the informational quality available for team decision-making is lower due to the greater degree of overlap in the information possessed by less diverse members (Williams and O’Reilly, 1998; van Knippenberg et al., 2004), and the benefits of the team reflexivity process may go unrealized.

As a unit-level and compositional construct, diversity is used to describe “the distribution of differences among the members of a unit with respect to a common attribute” (Harrison and Klein, 2007, p. 1200). The general perspective in the diversity literature is that diversity can occur around any number of factors (e.g., race, family background, functional background, immigration status, psychological traits and states) and that the differential status of individuals across these factors represents a diversity in the set of life experiences and resultant perspectives possessed by these individuals (Williams and O’Reilly, 1998; van Knippenberg and Mell, 2016). For the purposes of this study, we focus on “demographic” diversity (O’Reilly et al., 1989), also sometimes called “bio-demographic” diversity (Horwitz and Horwitz, 2007), as a proxy for diverse life experiences because it is stable (i.e., a “trait”) and readily measurable. However, the value of diversity does not lie in the variance across these demographic factors themselves but rather in the underlying diversity of perspective, experience, and information for which these demographic labels function as proxy.

Drawing on the information/decision perspective of diversity (Williams and O’Reilly, 1998; van Knippenberg et al., 2004; Meyer and Scholl, 2009), diverse groups are expected to possess a wider range of informational resources than homogenous groups, and such resources can contribute to their success if shared and integrated. High team diversity suggests team members have varied and unique knowledge, skills, and cognitive schemas (Harrison and Klein, 2007) related to their distinct backgrounds (e.g., education, family, function), which encourage members to view problems from different perspectives and generate different ideas, improving the quality of decision-making (Williams and O’Reilly, 1998; van Knippenberg et al., 2004). Because high levels of diversity mean that experiences are more likely to be unique, diverse perspectives can motivate members to carefully reflect on and share their experiences and then to deeply understand prior success and failures, which is beneficial for future team performance.

When teams have low diversity (i.e., possess homogenous information), not only is the quality of information processed through the reflexivity process lower, but the decision-making process itself may be hampered. Homogenous information is often associated with groupthink, and team members may regard consensus in decision-making as correctness (Chaiken and Stangor, 1987; Stasser and Birchmeier, 2003; Schulz-Hardt et al., 2006) and terminate the reflexivity process prematurely. However, when dissenting opinions are present, this signals that different viewpoints and information exist and merit consideration, discouraging use of a “consensus implies correctness” heuristic (Schulz-Hardt et al., 2006) and encouraging embrace of information sharing and the full reflexivity process. Research suggests that the presentation of diverse viewpoints supports processes that would improve the reflexivity process, such as stimulating divergent thinking (De Dreu and West, 2001), reducing confirmatory information search (Schulz-Hardt et al., 2000), and preventing groupthink (Smith et al., 1996).

For this study, we specifically focus on the demographic characteristic of family economic background diversity (i.e., the socioeconomic status in which participants grew up) in a Chinese context. Chinese society is marked by considerable socioeconomic stratification but also considerable mobility in the post-Mao period (Wu, 2019), allowing for the formation of work teams with members who grew up in households with distinctly different levels of affluence and resource availability. While under-studied in the management diversity literature, evidence suggests that socioeconomic status influences individuals’ perspectives and experiences in ways that affect their cognitions and behaviors later in life, including leadership behaviors (Barling and Weatherhead, 2016) and creative thinking (Eun et al., 2019). In a context marked by considerable social churn, we argue that the varied legacies of team members’ socioeconomic upbringings can be a source of heterogeneous information and perspectives that teams can draw upon when engaging in the reflexivity process.

Thus, we argue that team family economic background diversity contributes to the conditions necessary for teams to fully embrace the team reflexivity process and benefit from its positive effect on team decision quality and team performance (through decision quality). Under conditions of low team diversity, team members reflect on and process homogenous information, reducing the value provided by team reflexivity in terms of decision-quality and performance and possibly terminating the reflexivity process prematurely due to rapidly emerging consensus cutting off further sharing of the limited diverse information available for consideration.

Hypothesis 3: Team family economic background diversity moderates the relationship between team reflexivity and team decision quality, such that high team diversity reinforces the positive relationship between team reflexivity and team decision quality.

Hypothesis 4: Team family economic background diversity moderates the indirect relationship between team reflexivity and team performance through team decision quality, such that the indirect relationship is stronger when team diversity is high rather than low.

## Methods

### Sample and Procedure

We tested our hypotheses using a sample of CEOs and founding members of start-up companies in mainland China. We initially distributed questionnaires to 101 CEOs and 239 members in 101 entrepreneurship teams. A two-wave survey design was conducted to collect data. In the first survey (Time 1), team members were asked to rate their team’s reflexivity and provide their demographic information. Approximately 5 months later (Time 2), team members were asked to evaluate team decision quality, while CEOs assessed team (i.e., firm) performance and provided background information on themselves and the team. All questionnaires began with a simple introduction of the research purpose and the assurance of confidentiality. Individual IDs and team IDs were used to match the data for further analysis. The final sample includes 82 teams with 82 leaders and 194 team members. The average team size was 2.38 (SD = 0.70), and the average team age (years since company founding) was 2.46 years (SD = 0.81).

### Measures

The original English items were translated into Chinese by a scholar and translated back into English by another scholar to ensure equivalence (Brislin, 1980). All the survey items were responded to on a five-point Likert scale (1 = *strongly disagree* and 5 = *strongly agree*). Item scores of the same category were averaged to create an overall mean for each scale and coded such that high values represent high levels of the constructs.

#### Team Reflexivity

Team reflexivity was measured by a five-item scale developed by De Jong and Elfring (2010) with reference to Carter and West (1998) (α = 0.92). A sample item is “In the team, we often discuss the feasibility of our goals.”

#### Family Economic Background Diversity

Participants were asked about the economic background of their family of origin and responded using a five-point Likert scale (1 = *very poor* to 5 = *very rich*). We used CV coefficient (Allison coefficient of variation, e.g., Moynihan and Peterson, 2001) to calculate diversity. *CV* = SD/M ^∗^100%, where SD represents the standard deviation, M represents the mean.

#### Decision Quality

We measured decision quality by adapting a 6-item scale developed by Dooley and Fryxell (1999). A sample item is “My team makes decisions based on the best available information”.

#### Team Performance

As the sample comes from new venture, team performance was measured by the log of the sales growth rate of in the past 2 years.

#### Control Variables

We included four variables as controls: CEO gender, CEO educational attainment (1 = high school or vocational school graduate, 2 = junior college graduate, 3 = undergraduate, 4 = graduate, 5 = doctoral student or higher), CEO age (1 = below 25; 2 = 26−35; 3 = 36−45; 4 = 46−55; 5 = Above 56) and team founding time (measured by number of years), to preclude alternative explanations of the study findings.

## Results

We proposed a theoretical model at the team level, and team reflexivity and team decision quality were rated by every team member based using the consensus-based approach (Chan, 1998). Therefore, we calculated the within-group agreement indexes (rwg; James et al., 1993) and intraclass correlation coefficients [ICC (1) and ICC (2); Bliese, 2000] to confirm the reliability and validity of aggregating individual ratings. Results show a sufficient basis to support the aggregation of the two variables to the team level [Team reflexivity: *rwg* = 0.90, *ICC* (1) = 0.15, *p* = 0.04, *ICC* (2) = 0.30; Team decision quality: *rwg* = 0.92, *ICC* (1) = 0.28, *p* < 0.01, *ICC* (2) = 0.47].

Additionally, we conducted a confirmatory factor analysis (CFA) in AMOS 23.0 to examine the discriminant validity of the model. The theorized 4-factor model is a better fit [χ^2^ (61) = 97.097, *p* < 0.01, RMSEA = 0.055, CFI = 0.971, TLI = 0.962, IFI = 0.971, SRMR = 0.039] compared to the alternative models (see Table 1).

**TABLE 1:** Confirmatory factor analysis for discriminant validity.

Model	Factors	χ*2/df*	TLI	CFI	IFI	RMSEA	SRMR
Model 0	Four-factor model	1.592	0.962	0.971	0.971	0.055	0.039
Model 1	Three-factor model: combined TR and FEBD	1.672	0.957	0.965	0.966	0.059	0.044
Model 3	Two-factor model: combined TR, FEBD and TDC	8.006	0.554	0.628	0.632	0.191	0.183
Model 4	Two-factor model: combined TR and FEBD, TDC and TP	1.671	0.957	0.965	0.965	0.059	0.048
Model 5	One-factor model: combined all the four constructs	8.006	0.554	0.628	0.632	0.191	0.183

*TR = Team Reflexivity; FEBD = Family Economic Background Diversity; TDC = Team Decision Quality; TP = Team Performance. Although model 1 and model 4 also have good model fit results, we still choose model 0 because conceptually team reflexivity and family economic background diversity are distinct constructs. The similarity of model 0, 1 and 4 may result from the single-item measurement of family economic background diversity and team performance.*

Table 2 shows the means, standard deviations, reliabilities and correlations of the variables. Results show that team reflexivity is positively correlated with team decision quality (*r* = 0.25, p < 0.05), indicating a positive relationship between them. Further, Model 2 in Table 3 shows that team reflexivity is positively related to team decision quality (M2: *b* = 0.26, *p* < 0.05), thus supporting H1.

**TABLE 2:** Means, standard deviations, correlations, and internal consistency reliabilities.

	Mean	Std	1	2	3	4	5	6	7	8
1. Leader gender	1.27	0.45	1.00							
2. Leader education	3.24	0.84	0.05	1.00						
3. Leader age	3.05	0.66	–0.13	–0.04	1.00					
4. Team founding time	2.50	0.76	−0.21^†^	0.17	0.06	1.00				
5. Team reflexivity	3.99	0.35	0.18	–0.21	0.04	−0.24^∗^	(0.92)			
6. Team decision quality	3.90	0.37	0.05	0.07	–0.08	–0.09	0.25^∗^	(0.85)		
7. Family economic background diversity	0.23	0.17	–0.01	0.03	–0.13	–0.10	0.02	0.02	1.00	
8. Team Performance	1.38	0.41	0.05	0.22^∗^	0.06	–0.04	–0.01	0.22^∗^	–0.22	1.00

**N* = 82. Values in parentheses are reliability coefficients. ^∗^*p* < 0.05, ^†^*p* < 0.1 (two-tailed).*

**TABLE 3:** Regression results for mediation and moderation.

Variables	Team decision quality			Team performance			
	M1	M2	M3	M3	M4	M6	M7
Intercept	0.09 (0.32)	0.10 (0.31)	−0.02(0.31)	0.92 (0.35)**	1.07(0.35)^†^	0.33 (0.34)	−0.14 (0.34)
**Control variables**							
Leader gender	0.02 (0.10)	−0.01 (0.10)	0.02 (0.09)	0.03 (0.10)	0.03 (0.10)	0.03 (0.10)	0.01 (0.10)
Leader education	0.04 (0.05)	0.03 (0.05)	0.04 (0.05)	0.12 (0.05)*	0.12 (0.06)*	0.11 (0.05)	0.11 (0.05)*
Leader age	−0.04 (0.06)	−0.05 (0.06)	−0.03 (0.06)	0.05 (0.07)	0.05 (0.07)	0.06 (0.07)	0.04 (0.07)
Team founding time	−0.05 (0.06)	−0.02 (0.06)	−0.01 (0.06)	−0.04 (0.06)	−0.04 (0.06)	−0.04 (0.06)	−0.05 (0.06)
**Independent variables**							
Team reflexivity (TR)		0.26 (0.12)*	0.24 (0.12)*		−0.04 (0.13)	−0.10 (0.13)**	−0.10 (0.13)
**Mediator**							
Team decision quality						0.25 (0.12)*	0.30 (0.13)*
**Moderator**							
Family economic background diversity (FEBD)			0.10 (0.24)				−0.60 (0.26)*
**Interaction**							
TR × FEBD			1.64 (0.62)*				−0.99 (0.70)
*R*^2^	0.020	0.074	0.155^†^	0.062	0.063	0.111	0.183^†^
Δ*R*^2^		0.054^∗^	0.081^∗^		0.001	0.048^∗^	0.073^∗^

**N* = 82; Standard errors are reported in parentheses. ^∗∗^*p* < 0.01, ^∗^*p* < 0.05, ^†^*p* < 0.10 (two tailed).*

Model 5 in Table 3 shows that team decision quality was positively related to team performance (M5: *b* = 0.25, *p* < 0.05), controlling for team reflexivity and demographic variables. We also conducted a bootstrapping method analysis to examine the indirect effect of team decision quality. Results show that the indirect effect is significant (*b* = 0.06, 95% CI = [0.01, 0.19], not containing zero). Hence, H2 received support. Team decision quality partially mediates the relationship between team reflexivity and team performance.

**FIGURE 2:**
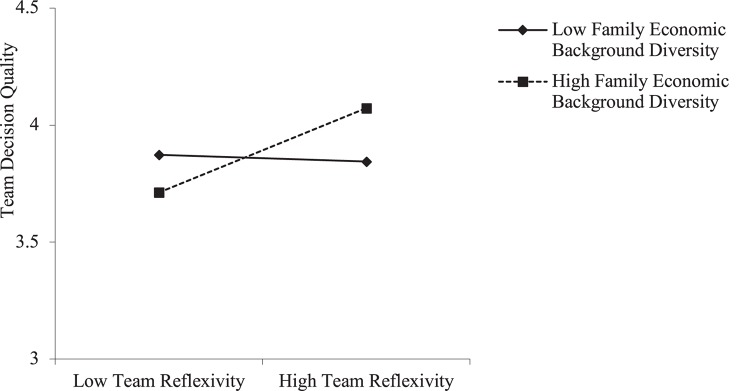
Moderating effects of family economic background diversity on the relationship between team reflexivity and team decision quality.

Hypothesis 3 predicts that team family economic background diversity positively moderates the relationship between team reflexivity and team decision quality. As shown in Table 3, the interaction of team reflexivity and family economic background diversity is significantly related to team decision quality after controlling for other irrelevant variables (Model 3: *b* = 1.64, *p* < 0.05). In addition, the interactive effect figure (Figure 2) and simple slope test indicate that, for teams with high family economic background diversity (M+1*SD*), team reflexivity is positively related to decision quality (*t* = 1.94, *p* = 0.057), but for teams with low family economic background diversity (M−1*SD*), team reflexivity is not significantly related to team decision quality (*t* = −0.33, *ns*). The difference between the two slopes is significant (*t* = 2.27, *p* < 0.01). This evidence supports H3.

Using the method laid out by Preacher et al. (2007), we examined whether there is a different conditional indirect effect of team reflexivity on team performance via team decision quality (H4). Drawing from a bias correction confidence interval derived from 5000 bootstrapped samples, results shows that, when family economic background diversity is high, the indirect effect of team reflexivity is significantly positive (*b* = 0.17, 95% CI = [0.02, 0.31], not containing zero). In contrast, when family economic background diversity is low, the indirect effect was not significant (*b* = −0.13, 95% CI = −1.14, 0.43], containing zero). Taken together, the hypothesized moderated mediation model (H4) received support.

## Discussion

Results from our study provide evidence in support of our model, especially relating to the underlying process of team decision quality through which team reflexivity affects team performance and the moderation of the reflexivity-decision quality and mediation relationships by team diversity. Our findings suggest that in teams where members have diverse family economic backgrounds, team reflexivity can be beneficial to team performance by improving team decision quality. However, when teams have low family economic background diversity, team reflexivity has no significant relationship with team decision quality and team decision quality does not significantly mediate the team reflexivity-performance relationship. The results of our moderated mediation model suggest that team performance is not the proximal outcome of team reflexivity and that reflexivity-performance relationship is complex and additional mediators and moderators merit future consideration.

### Theoretical Implications

This study makes several contributions to the team reflexivity literature and our understanding of how to meaningfully affect team performance. First, we empirically support the idea that team decision quality mediates the relationship between team reflexivity and team performance, serving as a more proximate outcome of team reflexivity and an explanatory mechanism. Previous work has examined shared task understanding and team learning as mediating mechanisms (e.g., van Ginkel and van Knippenberg, 2009; Schippers et al., 2013), and our exploration of the role of team decision quality builds on this work. We employ the information processing perspective (Schippers et al., 2014) and view team reflexivity as a systematic information processing approach, positing that the intentional information sharing and reflective approach in reflexivity can reduce information processing failures and support teams in making higher quality decisions and improving their performance. Because team decision quality is a more proximate outcome of team reflexivity but is still closely related to team performance, employing it in the study of team reflexivity allows us to parse out additional team and contextual elements that may also affect performance to better understand the effects of team reflexivity.

Second, we further contribute to the team reflexivity literature by identifying an additional important boundary condition for its effects on performance: team diversity. Previous literature has, at times, ignored the complexity of the reflexivity-performance relationship and the potential for the time and effort involved in the reflective process to overwhelm its positive effects resulting in a net negative effect on performance (Moreland and McMinn, 2010; Schippers et al., 2015; Konradt et al., 2016). Recent work has begun to investigate potential boundary conditions and identify situations in which the negative effect of the requisite time and effort is stronger than the positive effect of the reflection or in which the reflection itself is simply not helpful (e.g., Schippers et al., 2013, 2015). We advance this stream of research by identifying the moderating role of team diversity based on the information/decision perspective in which the diversity of information in a team, if shared, is thought to support team performance and decision-making (Williams and O’Reilly, 1998; van Knippenberg et al., 2004). When teams possess heterogeneous information because of their distinct backgrounds and experiences, their performance and decision-making can benefit from the multiplicity of perspectives and critical and integrative thinking required to contend with this heterogeneity during decision-making (Williams and O’Reilly, 1998; van Knippenberg et al., 2004). The presence of heterogeneous information can serve as a signal that deep reflection and engagement with the decision, rather than mere consensus, is required (Stasser and Birchmeier, 2003; Schulz-Hardt et al., 2006), and it increases the potential payoff of reflexivity processes in the form of increased team decision quality and performance.

### Managerial Implications

Our findings can also help managers better utilize team reflexivity as a tool to encourage team performance. First, the current study suggests that team reflexivity cannot always improve team performance, which encourages practitioners to consider the circumstances of their team (e.g., team members’ level of diversity and distributed information) when determining whether to direct members to reflect and share – managers need to monitor whether a team possesses informational resources that a potential reflexivity process could draw into the team’s decision-making. Second, when practitioners encourage team reflexivity, they should monitor the quality of the discussion and reflection to determine whether the process is likely to net the team performance benefits. Third, where possible, managers should encourage members to openly express their viewpoints and share their unique experiences rather than focusing on already-shared information or reaching consensus quickly.

Fourth, given the moderating effect of team diversity on team reflexivity-performance relationship, practitioners should pay attention to team members’ diversity when constructing teams that will engage in complex problem-solving and decision-making. To be specific, practitioners should arrange individuals with different backgrounds in one team to ensure team diversity. As previous research suggests, diversity is beneficial for team information processing and team innovation because it allows members to supply different ideas and perspectives (Williams and O’Reilly, 1998). Our results suggest that the benefit of this diversity is particularly pronounced in situations where teams are encouraged to reflect deeply and intentionally on their experiences, goals, and past performance. Some work suggests that team diversity can have negative effects (e.g., by inducing team conflict; Curseu and Schruijer, 2008; Mello and Delise, 2015); thus, managers must balance the informational benefits of diversity against the benefits of harmony that can arise from more homogenous teams.

Finally, team decision quality is an important proximal outcome of team reflexivity and process through which team reflexivity improves team performance. Managers may benefit from monitoring team decision quality rather than more distal indicators of team performance when determining whether a team reflexivity process is effective. As indicated by the decision quality literature, team members’ behaviors, such as interpersonal adaptability, flexibility, assertiveness, and communication quality, are positively related to team decision quality (Carmeli and Schaubroeck, 2006). Given this, managers are advised to encourage team members to collect diverse information and exchange information freely, train team members to be flexible, assertive, and adaptable, and promote team reflexivity.

### Strengths, Limitations and Future Research Directions

This study has several desirable features. First, our measure of family background diversity was derived from objective data. Second, we utilized multiple sources and multiple timepoints in collecting our data. Data on the independent variable (i.e., team reflexivity) was collected from team members several months before they were asked to rate their decision quality, and data on team performance was collected from an additional source, the team leader (i.e., CEO). The multi-source and multi-time nature of our research design helps reduce common method bias issues and can support conclusions about the causal nature of the relationship (Holland, 1986).

Despite this, we should note some limitations of our study that may inspire future research. First, we paid little attention to the temporal nature of team reflexivity. As pointed out by Konradt et al. (2016), team reflexivity takes place across an episodic cycle that is separated into transition and action phases. That is to say, team members reflect upon team-related information (i.e., states, procedures, and outputs) from previous performance episodes, and then prepare for future tasks (LePine et al., 2008; Schippers et al., 2013). In order to deal with the vital question of temporal issues, scholars should track multiple episodic cycles of reflexivity in future research.

Additionally, based on the information-processing perspective and information/decision perspective on diversity, we examined team decision quality and team diversity as the mechanism and boundary condition in the team reflexivity-performance relationship. Our findings suggest that team reflexivity is not a universally positive process and support the contention that the team reflexivity-team performance relationship is complex (Moreland and McMinn, 2010; Schippers et al., 2013). Other possible mediators or moderators should be studied to further explore the nuances of the reflexivity-performance relationship in the future.

Finally, while most studies have discussed the positive outcomes of team reflexivity, such as better performance, innovation, and employee wellbeing (Schippers et al., 2015; Chen et al., 2018), exploring the dark-side of team reflexivity and identifying its mechanisms is a constructive direction for future research to address the inconsistent reflexivity-performance relationship. For instance, studies have shown that team reflexivity is usually accompanied by resource depletion (e.g., time resource, cognitive resource; Schippers et al., 2013). Thus, scholars can also focus on the negative effect of team reflexivity on team performance by examining the mediating role of ego depletion in future research.

## Conclusion

Drawing on an information-processing perspective and information/decision theory, this study uses a field sample to investigate the relationship between team reflexivity and team performance. Our findings reveal that team reflexivity cannot predict team performance alone, and team diversity should be taken into consideration. To be specific, team diversity interacts with team reflexivity to positively influence team performance through the mediating mechanism of team decision quality. We hope that this study encourages interest in and focus on the quality of team reflexivity instead of just emphasizing frequent team reflexivity and spurs future examination of the complexities of the effect of team reflexivity on team performance.

## Data Availability Statement

The raw data supporting the conclusions of this artilce will be made available by the authors, without undue reservation, to any qualified researcher.

## Ethics Statement

The research has been performed in accordance with the recommendations of the Tsinghua University. No unethical behaviors existed in the research process. In the first part of the electronic questionnaire, we informed participants about the objectives of the study and guaranteed their confidentiality and anonymity. They were completely free to join or drop out the survey. We obtained oral and informed consent from every participant before fulfilling the survey.

## Author Contributions

MY and ZZ theorized and wrote the manuscript. HS contributed to the theory building and revised the manuscript. YL contributed to the data analysis. WC and ND collected the data and contributed to the literature review.

## Conflict of Interest

The authors declare that the research was conducted in the absence of any commercial or financial relationships that could be construed as a potential conflict of interest.
